# Prognostic significance of systemic immune-inflammation index in patients with nasopharyngeal carcinoma: a meta-analysis

**DOI:** 10.1186/s13643-022-02123-y

**Published:** 2022-11-19

**Authors:** Zesheng Zeng, Shengen Xu, Dingting Wang, Gang Qin

**Affiliations:** Department of Otolaryngology Head and Neck Surgery, the Affiliated Hospital of Southwest Medical University, Luzhou, 646000 China

**Keywords:** Systemic immune-inflammation index, Meta-analysis, Prognosis,·Nasopharyngeal carcinoma

## Abstract

**Background:**

Previous studies have investigated the prognostic value of the systemic immune-inflammation index (SII) in nasopharyngeal carcinoma (NPC). However, the results have been inconsistent. Therefore, this study aims to investigate the prognostic significance of SII in NPC through a meta-analysis.

**Methods:**

The PubMed, Web of Science, Embase, and Cochrane Library databases were thoroughly searched. The pooled hazard ratios (HRs) with 95% confidence intervals (CIs) were calculated to evaluate the prognostic value of the SII for survival outcomes.

**Results:**

A total of six studies comprising 2169 patients were included in the meta-analysis. Pooled analyses indicated that a high SII was significantly associated with worse overall survival (OS) (*HR* = 1.69, 95% *CI* = 1.36–2.09, *P* < 0.001) and progression-free survival (PFS) (*HR* = 1.60, 95% *CI* = 1.29–1.98, *P* < 0.001) in patients with NPC. Subgroup analysis showed that SII was a significant prognostic marker for PFS but not for OS in NPC.

**Conclusion:**

Our meta-analysis demonstrated that a high SII could be an efficient prognostic indicator of OS and PFS in NPC. In our opinion, SII could be used to predict long-term and short-term outcomes in patients with NPC. Furthermore, we suggest that SII be applied to help individual patients with NPC assess the prognostic risk.

**Systematic review registration:**

PROSPERO CRD42022321570

## Background

Nasopharyngeal carcinoma (NPC) is an epithelial carcinoma originating from the lining of the nasopharyngeal mucosa and is commonly observed at the pharyngeal recess (fossa of Rosenmüller) [1]. According to the International Agency for Research on Cancer (IARC), there were 133,354 new cases of NPC worldwide in 2020, accounting for 0.7% of all cancers diagnosed in the same period [2]. The geographical distribution of NPC is mainly in East and Southeast Asia, with approximately 77% of NPC incidences found in these areas [3]. Radiotherapy is the primary treatment approach for non­metastatic NPC [4]. Meanwhile, with the application of intensity-modulated radiotherapy (IMRT), concurrent chemoradiotherapy (CCRT), and concurrent chemoradiotherapy (CCRT), the overall prognosis of NPC has been dramatically improved [5–7]. However, distant failure is a crucial problem, and the treatment outcome of metastatic NPC remains unsatisfactory [8]. Although the American Joint Committee on Cancer (AJCC) tumor-node-metastasis (TNM) classification is commonly used to select treatment strategies and predict outcomes, it is still a challenge to predict the prognosis for individual patients accurately [8]. Consequently, developing new and specific prognostic biomarkers for the individualized treatment of patients with NPC is of great significance.

Systemic inflammation has been implicated in the pathogenesis and progression of cancer, and many inflammatory biomarkers have been reported as prognostic factors in various carcinomas [9, 10]. The systemic immune-inflammation index (SII) has been shown to function as an effective indicator of cancer patients’ inflammatory status, which is calculated using the following formula: platelet count × neutrophil count/lymphocyte count [11, 12]. Numerous studies have demonstrated that SII is related to the prognosis of solid tumors, such as hepatocellular carcinoma [11], esophageal squamous cell carcinoma [13], gastric cancer (GC) [14], pancreatic cancer [15, 16], breast cancer [17], lung cancer [18, 19], oral cancer [20], and other cancers. Several recent studies also showed the SII to b prognostic for NPC [21–26], although the results were inconsistent. For instance, in some studies [21–23], SII was reported as an independent prognostic factor for NPC; however, in other studies [24–26], SII was not documented as an independent prognostic factor for NPC. Therefore, this study aims to comprehensively explore the prognostic value of SII in patients with NPC through a meta-analysis.

## Materials and methods

### Study guidelines

The present study was performed following the Preferred Reporting Items for Systematic Reviews and Meta-Analyses (PRISMA) guidelines [27], and it has been registered with PROSPERO (CRD42022321570).

### Literature search strategies

Two researchers (Z. Z. and S. X.) independently carried out a systematic online literature search and data extraction. Our search covered PubMed, Embase, Web of Science, and the Cochrane Library databases from its inception through April 17, 2022, for articles that explored SII’s prognostic value for NPC. The following search terms were used: “nasopharyngeal carcinoma,” “nasophary*,” “systemic immune-inflammation index,” “systemic immune inflammation index,” and “SII,” and all searches were performed using a combination of MeSH terms and free-test words. The references of the included studies and reviews were carefully examined to identify eligible studies.

### Inclusion and exclusion criteria

The inclusion criteria were as follows: (1) the association between SII and the prognosis of patients with NPC was depicted; (2) SII was measured before clinical treatments; (3) an optimal cutoff value of SII was included; (4) outcomes of interest, including overall survival (OS), progression-free survival (PFS), or distant metastasis-free survival (DMFS), were reported; (5) the articles had sufficient data to evaluate the hazard ratio (HR) and 95% confidence interval (CI) of survival; and (6) studies published in English. Additionally, the exclusion criteria were as follows: (1) studies on cell lines, tissues, or animals, (2) studies reporting continuous variables for SII, and (3) studies in which the publication type was case series, review article, letter, editorial, or commentary.

### Data extraction and quality assessment

Two researchers (Z. Z. and S. X.) independently extracted data from each eligible publication, and discrepancies were resolved by discussion with a third investigator (D. W.). The extracted data included the following study information: first author, publication year, sample size, metastatic status, World Health Organization (WHO) histological type, American Joint Committee on Cancer (AJCC) stage, the cutoff value of SII, survival endpoints, follow-up time, age characteristics of patients, treatment methods, HRs, and the corresponding 95% CIs. This meta-analysis used OS as the primary endpoint, while PFS and DMFS were secondary endpoints. In this meta-analysis, HRs and the corresponding 95% CIs for prognosis were evaluated in two ways. First, HRs were acquired directly from the papers that have reported the HRs and 95% CIs in univariate and/or multivariate analysis. HRs in multivariate analysis were preferred because they improve precision in interpreting confounding factors. Second, HRs and 95% CIs were calculated from the survival curves using Engauge Digitizer version 11.1 if a study provided only Kaplan–Meier curves.

The quality of the included studies was evaluated using the Newcastle–Ottawa scale (NOS) [28] by two independent authors (Z. Z. and S. X.) in three aspects: selection, comparability, and exposure. The NOS scores range from 0 to 9. Studies with a NOS score of 6 or more were considered high-quality studies. When the results were inconsistent, agreements were reached through discussion with a third investigator (D. W.).

### Statistical analysis

This meta-analysis was performed using Review Manager (RevMan) software, version 5.3 (the Nordic Cochrane Center, Cochrane Collaboration, Copenhagen, Denmark). Summary statistics were performed using standard meta-analysis methods, with HRs used as an effective measure to assess the association between SII and prognosis in patients with NPC. Between-study statistical heterogeneity was determined using the Higgins *I*^2^ statistic and Cochran’s Q test, with data followed by *P* < 0.10 and/or *I*^2^ > 50% considered significant heterogeneity. In our study, a fixed-effect model (FEM) was adopted for some cases with significant homogeneity, while a random-effect model (REM) was performed for others. Since fewer than 10 articles were included, the use of funnel plots for publication bias detection was avoided [29]. A leave-one-out strategy was used for sensitivity analysis to determine the stability and reliability of the results. *P* < 0.05 was considered statistically significant.

## Results

### Search results

**Fig. 1:**
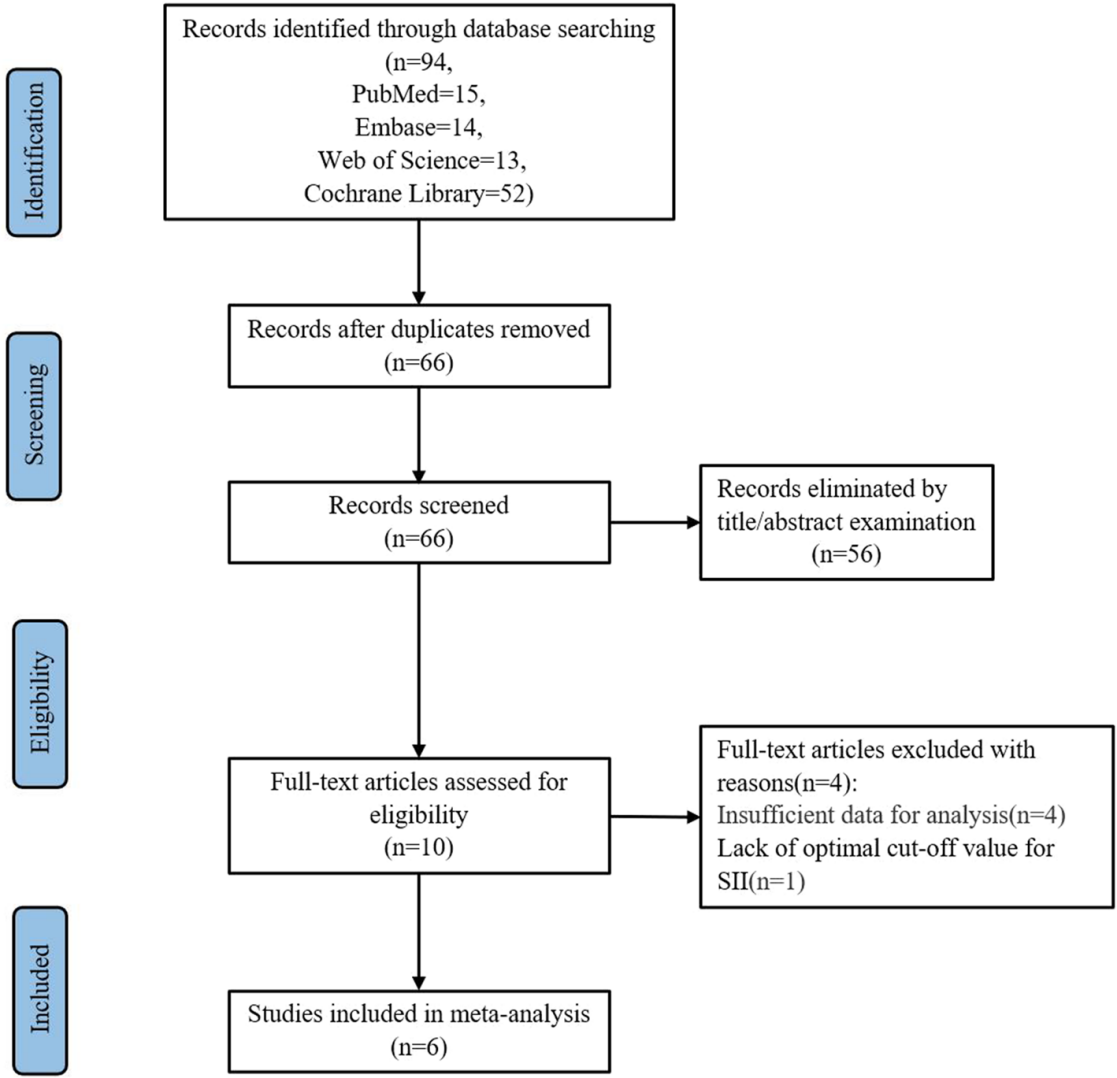
Flow chart of study selection

Through the use of search strategies, we located 94 studies in databases. After excluding duplicates, 66 studies remained. Afterward, 56 records were eliminated based on evaluations of the title and abstract, leaving ten studies for further evaluation. Following this, four studies were excluded for the following reasons: three lacked the necessary data for analysis, and another lacked an optimal cutoff value for SII. Ultimately, six studies [21–26] were included in the current meta-analysis with 2169 patients. The process of literature selection is described in Fig. 1.

### Study characteristics

The included studies were published between 2017 and 2021, with sample sizes ranging from 243 to 1767. All of them were conducted in China. All studies [21–26] enrolled patients with primary NPC. The prognostic effect of SII for OS was reported in all studies and was the primary endpoint of this meta-analysis. Moreover, four studies [22–24, 26], and one study [22], examined the relationship between SII and PFS and SII and DMFS, respectively. The median value of the cutoff for SII was 621.47 in the included studies. All included studies had NOS scores ≥ 6. A detailed description of all included studies is presented in Table 1.

**Table 1:** Characteristics of the included studies for the meta-analysis

First author	Year of publication	Patients (n)	Metastatic status	WHO histological type	AJCC stage	Cutoff value	Endpoints	Median follow-up (months)	Age (years)	Treatment	NOS score
Jiang	2017	327	Mixed	NA	I-IV (7th)	403.00	OS	38.3 (2–164.6)	50 (20–80)	Radiotherapy +/− chemotherapy	7
Oei	2018	585	Mixed	II/III	I-IV (7th)	527.20	OS/PFS/DMFS	63.3 (4.8–86.4)	49 (17–82)	IMRT	8
Lin	2019	243	Metastatic	I/II/III	NA	930.00	OS/PFS	77 (2–135)	48 (17–81)	Radiotherapy +/− chemotherapy	8
Feng	2020	417	Metastatic	NA	III-IV (8th)	488.90	OS/PFS	NA	47 (14–81)	Radiotherapy +/− chemotherapy	8
Zeng	2020	255	Mixed	I/II/III	I-IV (7th)	715.74	OS	33.5 (2.1–151.2)	51 (12–78)	Radiotherapy +/− chemotherapy, chemotherapy, untreated	6
Li	2021	342	Mixed	NA	I-IV (8th)	804.08	OS/PFS	66 (3–110)	49 (16-83)	IMRT, CCRT +/− induction, and/ or adjuvant chemotherapy	7

Abbreviations: *OS* Overall survival, *DMFS* Distant metastasis-free survival, *PFS* Progression-free survival, *WHO* World Health Organization, *AJCC* intensity-modulated radiotherapy, *NOS* Newcastle–Ottawa scale, *NA* Not available, *IMRT* Intensity-modulated radiotherapy, *CCRT* Concurrent chemoradiotherapy

### The association between SII and OS/PFS/DMFS in NPC

**Fig. 2:**
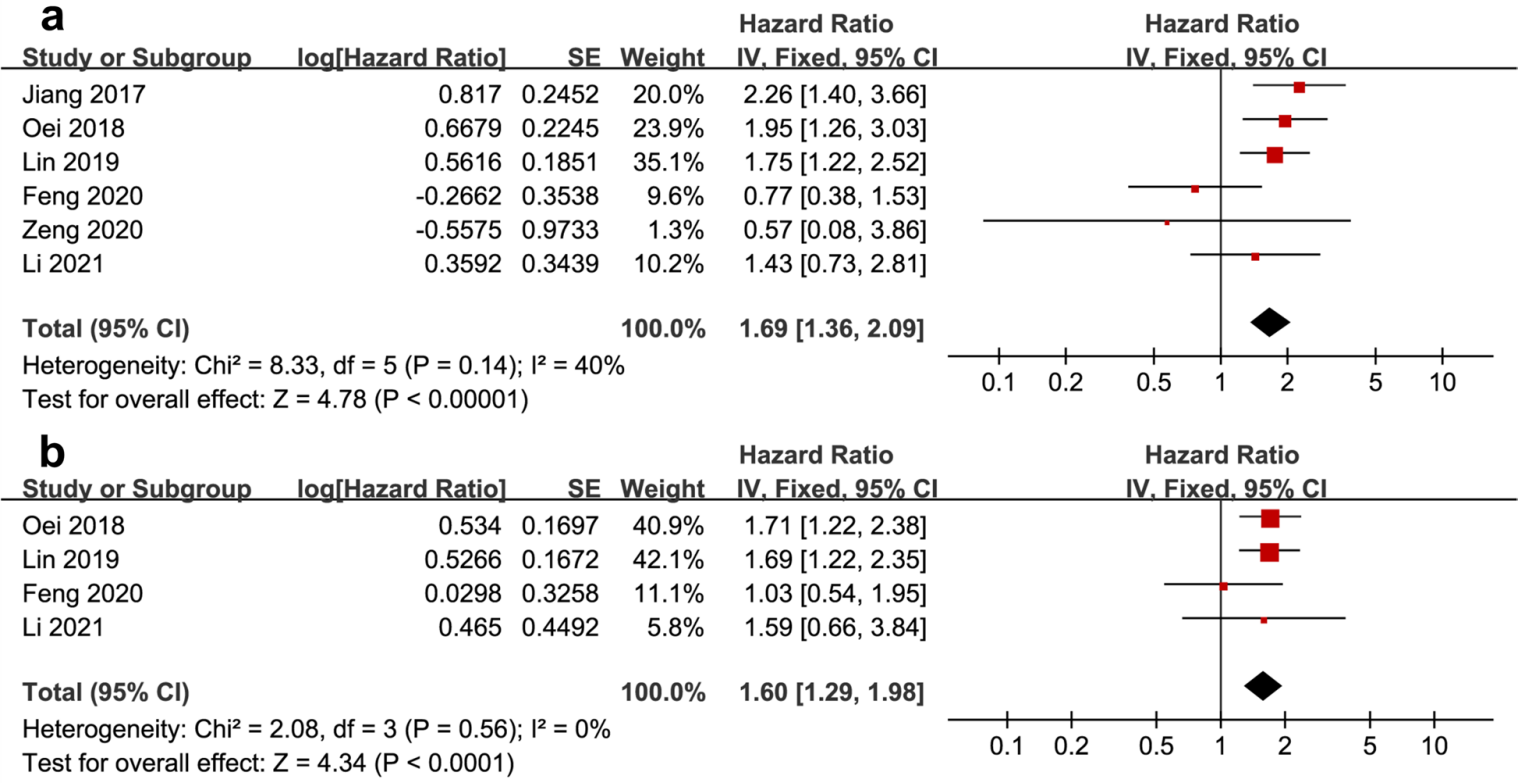
Forest plots reflecting the association between SII and OS (**a**)/PFS (**b**) in NPC. SII, systemic immune-inflammation index; *PFS*, progression-free survival

In total, six studies provided data for the calculation of the pooled HR and 95% CI of OS [21–26]. As a result of nonsignificant heterogeneity (*I*^2^ = 40%, *P* = 0.14), the FEM was applied. As shown in Fig. 2a, the pooled HR and 95% CI for OS was *HR* = 1.69, 95% *CI* = 1.36–2.09, and *P* < 0.001. In addition, the pooled HR and 95% CI of PFS were calculated based on data from 4 studies [22–24, 26]. As with the former, there was no significant heterogeneity (*I*^2^ = 0%, *P* = 0.56), and the FEM was applied. The pooled results for PFS are *HR* = 1.60 and 95% *CI* = 1.29–1.98, *P* < 0.001 (Fig. 2b). In addition, only one study [22] reported the prognostic value of SII for DMFS in NPC, and the results showed that SII was an independent prognostic factor for DMFS (*HR* = 2.089, 95% *CI* = 1.310–3.331, *P* = 0.002).

### Subgroup analyses

The OS and PFS survival outcomes were analyzed in subgroups. As shown in Table 2, a high SII was still a significant prognostic factor for OS in subgroups with a cutoff value > 527.2 and a sample size ≤ 327 and treated by IMRT (all *P* < 0.05). However, the association between SII and OS was not significant in the subgroups of cutoff value ≤ 527.2, sample size > 327, treatment except IMRT, and metastatic status (all *P* ≥ 0.05). In addition, high SII remained a significant prognostic factor for PFS, in all subgroups of cutoff value, sample size, treatment with radiotherapy +/− chemotherapy, and metastatic status (all *P* < 0.05).

**Table 2:** Subgroup analysis for OS and PFS in this meta-analysis

Subgroups	Number of studies	Number of cases	HR (95% *CI*)	*p*-value	Heterogeneity	
					*I*^2^ (%)	*p*-value
OS						
Overall	6	2169	1.69 (1.36, 2.09)	< 0.001	40	0.14
Cutoff value						
≤ 0.2	3	1329	1.59 (0.91, 2.78)	0.110	71	0.03
> 527.2	3	840	1.63 (1.19, 2.23)	0.002	0	0.48
Sample size						
≤ 327	3	825	1.87 (1.41, 2.49)	< 0.001	9	0.33
> 327	3	1344	1.35 (0.79, 2.33)	0.270	60	0.08
Treatment						
Radiotherapy +/− chemotherapy	3	987	1.54 (0.92, 2.59)	0.100	69	0.04
IMRT	1	585	1.95 (1.26, 3.03)	0.003	-	-
Radiotherapy +/− chemotherapy, chemotherapy, untreated	1	255	0.57 (0.08, 3.86)	0.570	-	-
IMRT, CCRT +/− induction, and/ or adjuvant chemotherapy	1	342	1.43 (0.73, 2.81)	0.300	-	-
Metastatic status						
Metastatic	2	660	1.22 (0.55, 2.74)	0.620	77	0.04
Mixed	4	1509	1.89 (1.42, 2.52)	< 0.001	0	0.44
PFS						
Overall	4	1587	1.60 (1.29, 1.98)	< 0.001	0	0.56
Cutoff value						
≤ 432.48	2	1002	1.53 (1.14, 2.06)	0.005	47	0.17
> 432.48	2	585	1.68 (1.24, 2.28)	< 0.001	0	0.90
Sample size						
≤ 342	2	585	1.68 (1.24, 2.28)	< 0.001	0	0.90
> 342	2	1002	1.53 (1.14, 2.06)	0.005	47	0.17
Treatment						
Radiotherapy +/− chemotherapy	3	1245	1.60 (1.29, 1.99)	< 0.001	4	0.35
IMRT, CCRT +/− induction, and/ or adjuvant chemotherapy	1	342	1.59 (0.66, 3.84)	0.300	-	-
Metastatic status						
Metastatic	2	660	1.53 (1.14, 2.04)	0.004	46	0.17
Mixed	2	927	1.69 (1.24, 2.31)	< 0.001	0	0.56

Abbreviations: *OS* Overall survival, *HR* Hazard ratio, *PFS* Progression-free survival, *CI* Confidence interval, *IMRT* Intensity-modulated radiotherapy, *CCRT* Concurrent chemoradiotherapy

### Sensitivity analyses

To assess the robustness of the results, a sensitivity analysis was performed. The tests showed no significant change in the pooled results for OS and PFS when a single trial was excluded. Therefore, we consider the evidence assembled in the current meta-analysis to be robust and credible.

### Publication bias

Due to the small number of studies included (*n* = 6), no publication bias test was performed.

## Discussion

To the best of our knowledge, this is the first meta-analysis to describe the prognostic significance of SII in NPC. The SII is a combined marker based on platelet, lymphocyte, and neutrophil counts [11], which could indicate the body’s inflammatory status effectively. In this meta-analysis, by collecting data from 2382 patients across six studies, we demonstrated that a high SII was a significant prognostic indicator for lower OS, PFS, and DMFS in NPC. Platelet, lymphocyte, and neutrophil counts are routinely measured by laboratory tests in NPC patients before treatment. We believe that SII is easily available and cost-effective in clinical practice. Therefore, we suggest that SII should be applied to help individual patients with NPC assess the prognostic risk.

Previous studies have shown that systemic inflammation influences the pathogenesis and progression of cancer [9, 10]. Firstly, platelets contribute to tumor cell proliferation and metastasis through direct interactions and secreted bioactive proteins [30]. In addition, tumor-associated neutrophils can exert protumoral functions, enhancing tumor cell invasion and metastasis, angiogenesis, and extracellular matrix remodeling while inhibiting antitumoral immune surveillance [9]. In contrast, lymphocytes commonly function as pivotal tumor suppressors by inducing cytotoxic cell death and producing cytokines that inhibit cancer cell proliferation and metastatic activity [31]. Therefore, a high SII indicator of poor prognosis is based on the different roles of platelets, neutrophils, and lymphocytes in tumor biology.

Recently, a series of meta-analyses have explored the prognostic significance of SII in various solid tumors [19, 32–36]. First, Zhang et al. [33] suggested that a high SII was related to poor prognosis in breast cancer patients and clinicopathological features that indicated tumor progression. Additionally, Li et al. [34] reported that SII could serve as a promising noninvasive biomarker to assess the prognosis of patients with urinary system cancer. Another meta-analysis showed that a higher pre-treatment SII was significantly associated with poorer survival outcomes and several clinical characteristics in GC [35]. A late meta-analysis of 11 studies recruiting 2365 patients demonstrated that SII was a significant prognostic marker for survival in patients with pancreatic cancer [36]. In addition, Wang et al. [32] illustrated that an elevated SII was a factor of poor prognosis in patients with hepatocellular carcinoma. Furthermore, Zhou et al. [19] reported that a high SII could be an effective indicator of the prognosis of OS in SCLC. In this meta-analysis, pooled results indicated that NPC patients with high SII values had worse OS, PFS, and DMFS than those with low SII values. Moreover, subgroup analyses were applied to investigate the prognostic significance of SII in patients with different cutoff values, sample sizes, treatment modalities, and metastatic statuses. The findings showed that SII was not a significant prognostic marker for OS but for PFS in NPC. This may be due to the relatively small sample size. In addition, the SII value was associated with DMFS in NPC, although this result needs further validation.

It should be acknowledged that this meta-analysis has several limitations. First, the six included articles were retrospective cohort studies, which may have inherent limitations. Second, the total number and sample size included in the study were relatively small, especially in some subgroup analyses. Therefore, it is necessary to carry out more large sample studies to further verify these findings. Third, due to the lack of original data from individual studies, subgroup analyses based on other factors, such as age, sex, and AJCC stage of the tumor, were not performed. Fourth, all the research results were from China, and there were limitations in their prognostic significance for patients with NPC in different countries. Finally, the optimal cutoff values of the SII were inconsistent in various studies. Given the limitations enumerated above, we should be cautious in interpreting the results of this study.

In conclusion, our meta-analysis demonstrated that a high SII was related to NPC’s poor OS, PFS, and DMFS. In our opinion, SII could be used to predict long-term and short-term outcomes in patients with NPC. Furthermore, we suggest that SII should be applied to help individual patients with NPC to assess the prognostic risk.

## Data Availability

All data generated or analyzed during this study are included in this published article (and its supplementary information files).

## Abbreviations

SII: Systemic immune-inflammation index
NPC: Nasopharyngeal carcinoma
HR: Hazard ratio
CI: Confidence intervals
OS: Overall survival
PFS: Progression-free survival
DMFS: Distant metastasis-free survival
GC: Gastric cancer
PRISMA: Preferred Reporting Items for Systematic Reviews and Meta-Analyses
RevMan: Review Manager
WHO: World Health Organization
AJCC: Intensity-modulated radiotherapy
NOS: Newcastle–Ottawa scale
NA: Not available
IMRT: Intensity-modulated radiotherapy
CCRT: Concurrent chemoradiotherapy
